# Understanding, Recognising and Treating Co-occurring Anxiety in Autism

**DOI:** 10.1007/s40474-018-0132-7

**Published:** 2018-01-23

**Authors:** Jacqui Rodgers, A. Ofield

**Affiliations:** 1 Clinical Psychology, Institute of Neuroscience, Sir James Spence Institute, Newcastle University, Royal Victoria Infirmary, Queen Victoria Road, Newcastle, NE1 4LP UK; 2 Northumberland, Tyne and Wear NHS Foundation, Newcastle upon Tyne, UK

**Keywords:** Autism spectrum disorder, Anxiety, Assessment, Mechanisms, Treatment

## Abstract

**Purpose of Review:**

Autistic people are at increased risk of anxiety, with around 50% of autistic adults and children experiencing this debilitating mental health condition. The purpose of this review is to consider some contemporary ideas about underlying mechanisms for anxiety in autism, explore issues in the identification and assessment of anxiety and discuss emerging trends in anxiety interventions for autistic people, before identifying some important next steps in the field.

**Recent Findings:**

Emerging evidence suggests that anxiety may present differently in autism compared to the general population and that whilst CBT holds promise, there may be important differences in neurobiological, affective and cognitive responses to stressors for autistic people, which warrant tailored anxiety models, assessments and interventions.

**Summary:**

We conclude that research is needed to develop and evaluate theoretical frameworks, assessment methods and interventions for anxiety in autism, particularly for autistic adults and those with co-occurring intellectual disability.

## Introduction

Autism spectrum disorder (ASD) is a lifelong neurodevelopmental condition characterised by deficits in social communication and restrictive and repetitive behaviours [1]. Sadly, autistic people are at increased risk of experiencing co-occurring mental health difficulties, compared to the general population. One of the most common mental health difficulties associated with ASD is anxiety [2••]. Significant co-occurring anxiety has been reported to be present for around 50% of autistic children and adolescents [3, 4], whilst Lugnegard, Hallerbäck and Gillberg [5] report anxiety to be present in 50% of a sample of autistic 20–38 year olds. Buck, Viskochil, Farley et al. [6] report that 56% of their sample of autistic adults met criteria for at least one psychiatric disorder, with anxiety disorder found to have the highest current and lifetime prevalence within the sample.

When anxiety is present for autistic individuals, it is often complex. Multiple anxiety disorders often occur concurrently, with symptoms likely having both an additive and an interactive impact. In addition, there is growing evidence that some of the symptoms of anxiety may manifest differently in autistic individuals [2••, 7]. Both issues pose particular challenges for autistic people and their families and for researchers and clinicians. These complicating factors mean we all must work much harder to gain an accurate understanding of the underlying mechanisms and phenomenology of anxiety in autism, in order to develop sensitive methods of recognising anxiety when it occurs and treating it effectively.

This review paper will consider some contemporary ideas about underlying mechanisms for anxiety in autism. We will also outline issues in the identification and assessment of anxiety in ASD. Finally, we will explore some emerging trends in anxiety interventions for autistic people. We suspect that what the reader may conclude when reading this review is that, whilst there has been an upsurge in interest and research activity in this topic over the last 10 years or so, there remains much more to do and so we will conclude our piece with some consideration of possible next steps.

## Mechanisms of Anxiety in ASD

Of course, good clinical and research practice needs to be underpinned by robust theoretical frameworks, to inform the questions we ask, the assessments we use and the interventions we deliver. A critical question that currently remains unanswered is ‘why are autistic individuals more vulnerable to anxiety and why, when it is present, does anxiety present differently to traditional conceptualisations’? In order to answer this question, we need to consider the potential mechanisms that may underpin anxiety presentations in ASD. This is tricky stuff given the heterogeneity of autism and the complexity of anxiety, but research is beginning to emerge to address this challenging issue. It is not possible in this piece to consider all possible mechanisms for anxiety in ASD. Here, we identify some interesting and contemporary trends within the research literature for the reader to consider.

Some research indicates that differences in brain morphology in autistic people in areas of the brain associated with anxiety, e.g. the amygdala, may underpin the onset of anxiety for some individuals. For example, Herrington, Maddox, Kerns et al. [8••] completed structural magnetic resonance imaging (MRI) investigations with 53 autistic children. Around half of the samples were experiencing anxiety whilst the remainder were not. A typically developing comparison group was also included. The autistic children who were experiencing anxiety had decreased right amygdala volume compared to the other two groups, who did not differ from each other. These findings suggest that autistic children who experience anxiety may have differing neurodevelopmental trajectories from autistic children who are not anxious and from children who are not autistic. These findings are important because they contribute to the debate about whether, given its almost ubiquitous nature, anxiety can be regarded as a distinct disorder co-occurring alongside autism for some autistic individuals or whether anxiety is simply a feature of, or manifestation of, autism. These data suggest the former that anxiety is a distinct clinical entity separable from autism. This in turn has important implications for the identification and treatment of anxiety in ASD.

Other researchers have investigated differences in how autistic people may respond in threatening situations, for example, South, Rogers and van Hecke [9] and Mertens, Zane and Neumeyer et al. [10] both recently reported that autistic children and adolescents showed attenuated skin conductance activity in threatening situations. What this reported reduction in physiological response in the face of an anxiety provoking situation means is not yet fully understood. It may be that autistic individuals actively and rapidly disengage from stimuli that are anxiety provoking because they are highly aversive. The consequence of this avoidance is that they are therefore affected less by the experience. Alternatively, it may be that these data illustrate an important interaction between autism characteristics and responses to anxiety. To elucidate, pre-existing deficits in the capacity to respond in a flexible manner to environmental stimuli (a hallmark of ASD) are exacerbated in situations of high arousal, resulting in what appears to be an attenuated physiological response [11].

The important take home message from these data is that there is emerging evidence that there may be altered neurobiological responses to stressors for anxious autistic individuals that differ from responses seen in typically developing individuals. This has implications for clinical work (and, of course, for research). One of the techniques we use to monitor our affective state is to ‘listen’ to our bodily signs and symptoms, noticing when we feel agitated or aroused. If autistic individuals are not experiencing the interoceptive cues as readily, then this has implications for how they will learn to recognise and monitor their affective states and engage in anxiety reducing behaviours. Difficulty in recognising and understanding ones’ own emotions (known as alexithymia) has been identified as a common difficulty in ASD, affecting 40–65% of individuals [12] and importantly has recently been reported as a key cognitive mechanism in the development on anxiety in autism [13••]. The reported neurobiological differences in responsivity may be at the root of these emotion recognition difficulties associated with ASD.

Another key mechanism that has recently been implicated in relation to anxiety in ASD is *intolerance of uncertainty* (IU). IU is a broad dispositional risk factor for the development and maintenance of anxiety, which involves the ‘tendency to react negatively on an emotional, cognitive, and behavioural level to uncertain situations and events’. The presentation of IU resonates clinically with some of the core characteristics of ASD, particularly the presence of restricted and repetitive behaviours, which may represent an attempt to impose predictability in an uncertain world [14, 15•, 16••]. Indeed, anecdotally, parents often report increases in their child’s repetitiveness or insistence on sameness when they are feeling anxious. In clinical settings, asking whether an autistic child or adult has increased their engagement in RRB (from their usual baseline) can act as a simple and effective screening question to indicate that further exploration of possible anxiety is warranted. Recent work suggests that IU may have a central role in the relationship between ASD and anxiety. Boulter, Freeston, South and Rogers [17] modelled the relationship between anxiety and IU in a group of autistic children and a neurotypical comparison group. Results confirmed significant relationships between IU and anxiety in the autistic group, which were consistent with a model suggesting that IU mediates the relationship between ASD and anxiety. Wigham, Rogers, South, McConachie and Freeston [18] then went on to examine the role that IU may have in relation to two ASD-related characteristics that have independently been associated with increased anxiety, namely sensory processing atypicalities and restricted and repetitive behaviours. These relationships were mediated by IU, indicating that IU may play a particular role in the interaction between anxiety and ASD traits, perhaps explaining some of the increased vulnerability to anxiety we see in autistic populations. This was further supported by Neill et al. [19] who reported that IU is an important construct to both sensory sensitivities and to anxiety in autistic children. Kerns et al. [20] in a discussion of the differential diagnosis of anxiety disorders in autism report that *fears associated with uncertainty* may be an important mechanism in the development and maintenance of anxiety in ASD. This evidence indicates that IU is a key mechanism in the development and maintenance of anxiety in autistic people.

## How Do We Identify Anxiety in ASD?

Traditionally, when a clinician wishes to assess an individual to determine whether they are experiencing anxiety, or when researchers wish to assess anxiety for a research study, they will utilise some form of symptom-based checklist based on the diagnostic criteria associated with the different anxiety sub-types (e.g. social phobia, separation anxiety). These symptom checklists, embedded within interviews or questionnaires, are based on well-established criteria outlined in diagnostic manuals such as DSM-5 or ICD 10, the assumption being that there will be homogeneity in the presentation of mental health difficulties by individuals with these conditions. This is typically the method that has been used to identify anxiety in autistic children and adults for research (and probably clinical) purposes, with well-established diagnostic and assessment tools validated with the general population delivered to autistic people without modification. However, there is growing evidence that, for at least some autistic individuals, anxiety may present somewhat differently from the ways in which it presents in typically developing individuals, questioning the validity and reliability of these ‘off the shelf’ tools. To illustrate, Kerns et al. [2••] report on anxiety characteristics that are either consistent (i.e. traditional) or inconsistent (i.e. ambiguous) with DSM-based definitions of anxiety in autistic children. Seventeen percent of their sample was found to present with ‘traditional’ symptoms of anxiety, whilst 15% presented with ambiguous anxiety and 31% with a mixture of the two. Similarly, Ollendick and White [21] reported that within their sample of autistic children, there was evidence for what they termed shared anxiety characteristics (i.e. symptoms that are common in typical samples also) and ASD-specific anxiety processes. In this context, shared processes included negative biases, unhelpful automatic thoughts and physiological arousal, whereas processes they identified as unique to ASD included social confusion, difficulties in reading emotions in others (alexithymia), sensory sensitivities, negative social interactions with others, rigidity and insistence on sameness.

Given this emerging evidence for the atypicality of anxiety in ASD, researchers have begun to explore the measurement properties of some commonly used anxiety assessment tools for use with autistic people. To date, this work has largely been undertaken with measures utilised with children. This growing evidence base indicates that some of the most commonly used questionnaire measures of anxiety, developed with typically developing children, are not robust when used with autistic children. For example, Glod, Honey, Riby and Rogers [22], Magiati, Lerh, Uljarević et al. [23] and Jitlina, Zumbo, Mirenda et al. [24] all examined the psychometric properties of the widely used Spence Children’s Anxiety Scale (SCAS) for use as a self- or parent report with autistic children. These three studies, with independent samples, all concluded that the measure does not entirely capture the essence of anxiety in ASD. Scahill, Hallett, Aman et al. [25] reached similar conclusions whilst investigating the appropriateness of the Child and Adolescent Symptom Inventory (CASI) with a large sample of parents of autistic children. For reviews of the measurement properties of anxiety measurement tools for use with autistic children, also see LeCavalier, Wood, Halladay et al. [26•] and Wigham and McConachie [27•].

In response to these growing concerns about the assessment of anxiety in ASD, new autism-specific measures are emerging. In 2016, we published the first-ever parent and self-report anxiety questionnaire for autistic children (The Anxiety Scale for Children – ASD: ASC ASD; Rodgers, Wigham, McConachie et al. [28•] see http://research.ncl.ac.uk/cargo-ne/) and work is currently underway to adapt the tool for use with autistic adults with and without intellectual disability. In a similar vein and building on their earlier work, Scahill, Hallett, Aman et al. [25] are currently working on the development and validation of a new measure of anxiety for autistic children. Kerns has also developed an autism addendum to the Anxiety Disorders Interview Schedule [ADIS-2]. These exciting developments in measurement will of course require careful monitoring over time with further validation work undertaken to ensure that these newly adapted or developed tools are fit for purpose.

## What Can We Do to Help?

The ultimate goal of all of this work, of course, is to try to reduce the impact that anxiety has on the lives of autistic people and those supporting them and so it is appropriate that we spend some time considering progress that has been made with regard to interventions. Over the last 10 years or so, evidence has begun to emerge that supports use of modified cognitive behaviour therapy (CBT) as an effective treatment approach to address anxiety in ASD [29–33] with generally moderate effect sizes [34•]. Traditionally, interventions have been delivered to families in clinical settings. However, some recent evidence suggests that alternate settings may be as efficacious. Clarke, Hill and Charman [35] and Luxford, Hadwin and Kovshoff [36] reported promising results following randomised controlled trials of Attwood’s (2004) *Exploring Feelings* CBT programme for anxiety in autistic children delivered in a school-based setting. Clarke, Hill and Charman [35] report significantly lower levels of anxiety in the intervention group compared to the control group, which were maintained at 6- to 8-week follow-up. Similarly, Luxford, Hadwin and Kovshoff [36] found positive change for parent-, teacher- and self-reported anxiety symptoms with effects maintained at 6-week follow-up. Drmic, Aljunied and Reaven [37] present similarly promising results for a school-based adaptation of the *Facing Your Fears* programme [38] delivered in Singapore. This study makes a particularly important contribution, as not only was the CBT protocol culturally adapted to meet the needs of autistic adolescents with ASD in Singapore, it was also delivered by non-clinicians. They reported a significant decrease in self- and parent-reported anxiety. These important contributions have implications for the feasibility of delivering interventions by professionals outside of a clinical context, potentially increasing the reach of treatment programmes to children who may not be able to access more traditional forms of healthcare provision.

We are also seeing exciting development in the utilisation of technology in the delivery of psychological therapies, including the use of virtual reality environment (VRE) to treat specific fears and phobias in autistic children [39]. Traditionally, CBT with graded exposure as key therapeutic techniques is the standard method to treat phobias. However, these techniques may be less effective for some autistic people because of the requirement to engage in imaginal desensitisation. Difficulties with imagination are common in autism and reduced capacity to create imaginal scenes may therefore be a barrier to treatment adherence and/or effectiveness. VRE settings, which include computer-generated scenes to facilitate graded exposure to the phobic stimuli without reliance of imagination, are a way to overcome this difficulty and have yielded some promising results with autistic children [39, 40] and adults [41].

Earlier in this review, we considered emerging evidence for neurobiological differences in response to anxiety provoking or threatening situations that have been reported in autistic children and adults [8••, 9, 15•], and how these differences might underpin some of the difficulties with emotion recognition and understanding that are a hallmark of ASD. Targeted emotional literacy training alongside mindfulness-based techniques may be useful to support autistic people (both children and adults) to develop the skills to assist them to recognise their emotional experiences more effectively, tolerate the experience of previously aversive emotions and respond flexibly to stressors, rather than falling back on avoidance as a strategy to manage affect [42]. Indeed, Cachia, Anderson and Moore [43•] conducted a systematic review of the literature for mindfulness-based interventions with individuals with ASD. Their findings suggested that mindfulness is potentially effective at promoting psychological well-being in individuals with ASD.

We also considered the role that intolerance of uncertainty (IU) may have in the development and maintenance of anxiety in autistic children and adults and it is important to revisit this cognitive construct when considering treatment. Keefer, Kreiser, Singh et al. [44] examined whether IU impacted on treatment response following modified CBT for anxiety, using a well-established manualised programme [‘Facing your Fears’: 38], with 43 autistic children. They report that higher levels of pre-intervention IU predicted higher anxiety and worry both pre- and post-intervention. Importantly, the manualised treatment programme utilised did not specifically target IU. They conclude that directly targeting IU may improve outcomes for autistic children with high anxiety. Building on the growing evidence for the central role that IU may have in anxiety in autism, our group have recently developed the first intervention programme specifically targeting IU in autistic young people: Coping with Uncertainty in Everyday Situations (CUES©: 45•). CUES© is a parent group intervention that provides parents of autistic young people with strategies to reduce IU in their children in everyday situations. Working through parents is appropriate because it provides parents with strategies that they can utilise with their child across a range of everyday contexts. It also supports generalisation of these strategies outside of the clinic setting, as well as countering well-meaning, understandable but ultimately unhelpful/counter-productive strategies used by parents to try to build certainty around the child, when certainty is not realistically possible. We report preliminary evidence that CUES© is feasible and acceptable to families, that parents found it helpful to work with clinicians to develop a range of strategies to tackle uncertainty in everyday contexts that they can support their child to utilise, and parents and children reported beneficial effects on everyday functioning. Work is continuing to evaluate the programme and to develop an adaption of CUES specifically for autistic adults [7].

## Conclusion

This report has been a bit of a whistle stop tour of some of the key emerging trends in anxiety research in autism. In many senses, the field has developed so much over the past 5 years that we are in the very positive position of having almost an *embarrassment of riches*, and certainly too much high-quality work to cover in a brief report such as this. We do hope that we have been able to whet the reader’s appetite for this field however, because whether you are a clinician or a researcher (or both), there is still much to be done. There are many aspects of this topic that are under researched or not well specified and we need to continue to work together to better understand the aetiology, maintenance factors, phenomenology and best ways to treat anxiety experienced by autistic people.

So what remains to be tackled? Strong research and evidence-based clinical practice is, by necessity, underpinned by robust theoretical frameworks, which guide assessment and measurement and inform interventions. As we have seen, CBT-based programmes for young autistic people experiencing anxiety are emerging and this is undoubtedly a positive step [29–33]. These intervention programmes have been variously adapted to meet the needs and learning styles of the population. However, the application of these techniques, driven by the increasing awareness of the mental health needs of this group, is often in advance of clear understanding of the underlying mechanisms inherent in anxiety in ASD. There remains much still to be done to specify models of anxiety for ASD populations to enable the development of more targeted and effective intervention programmes. Whilst models of anxiety relevant to autism are emerging [16••, 49], and informing treatments for children [45•] and adults [7], this nascent work requires evaluation and further empirical support. Similarly, whilst progress has been made in relation to the development of specific, tailored assessment methods [20, 25, 28••], further validation work is required to consolidate these tools and ensure that they are fit for purpose. In addition, it will not have escaped the reader’s notice that much of the work to date has been undertaken with children and with individuals who do not have co-morbid intellectual disability. Autism is a lifelong condition and yet we know very little about the developmental trajectory of anxiety into adulthood. Furthermore, around 50% of autistic individuals also have intellectual disability; many of whom will also experience mental health problems, including anxiety [46, 47]. Mental health services are currently woefully limited in their ability to provide effective interventions for autistic people with intellectual disability, and tailored interventions are needed for this much neglected group. Finally, we currently know very little about gender differences in emotional awareness, anxiety symptomatology and treatment response, although emerging work urges us to pay attention to these factors as we move forwards [48, 49].

In sum, we have come a long way in a relatively short time as a research and clinical community in our efforts to put mental health issues, and specifically anxiety, on the agenda for those in the autism community that we serve, but there remains much to be done. It is critical that this work continues to develop and that we include the views and expertise of autistic collaborators and researchers at every step of this journey.
